# Centrosome amplification arises before neoplasia and increases upon p53 loss in tumorigenesis

**DOI:** 10.1083/jcb.201711191

**Published:** 2018-07-02

**Authors:** Carla A.M. Lopes, Marta Mesquita, Ana Isabel Cunha, Joana Cardoso, Sara Carapeta, Cátia Laranjeira, António E. Pinto, José B. Pereira-Leal, António Dias-Pereira, Mónica Bettencourt-Dias, Paula Chaves

**Affiliations:** 1 Instituto Português de Oncologia de Lisboa Francisco Gentil, Lisbon, Portugal; 2 Instituto Gulbenkian de Ciência, Oeiras, Portugal; 3 Faculdade de Ciências da Saúde, Universidade da Beira Interior, Covilhã, Portugal; 4 Ophiomics-Precision Medicine, Lisbon, Portugal

## Abstract

Centrosome abnormalities are a typical hallmark of human cancers. However, the origin and dynamics of such abnormalities in human cancer are not known. In this study, we examined centrosomes in Barrett’s esophagus tumorigenesis, a well-characterized multistep pathway of progression, from the premalignant condition to the metastatic disease. This human cancer model allows the study of sequential steps of progression within the same patient and has representative cell lines from all stages of disease. Remarkably, centrosome amplification was detected as early as the premalignant condition and was significantly expanded in dysplasia. It was then present throughout malignant transformation both in adenocarcinoma and metastasis. The early expansion of centrosome amplification correlated with and was dependent on loss of function of the tumor suppressor p53 both through loss of wild-type expression and hotspot mutations. Our work shows that centrosome amplification in human tumorigenesis can occur before transformation, being repressed by p53. These findings suggest centrosome amplification in humans can contribute to tumor initiation and progression.

## Abstract (precis)

Lopes et al. examine centrosomes in human samples progressing from premalignant to metastatic lesions from patients with Barrett’s esophagus. They find that centrosome amplification can occur before transformation during human tumorigenesis, being repressed by p53, suggesting that centrosome amplification contributes to tumor initiation before p53 mutation.

## Introduction

The centrosome has key roles in microtubule organization, signaling, polarity, and cell division—all processes deregulated in tumorigenesis. Each centrosome, composed of two centrioles and a pericentriolar protein matrix (PCM), duplicates once per cell cycle to ensure bipolar spindle assembly during cell division (Bornens, 2012; Godinho and Pellman, 2014). Centrosome number amplification can lead to aberrant mitotic spindles and associated cell death (Holland et al., 2012; Marthiens et al., 2013). However, cancer cells with centrosome amplification can often survive cell division while generating genomic instability (Ganem et al., 2009; Silkworth et al., 2009). Moreover, centrosome amplification can promote aneuploidy and invasiveness in cultured cells as well as promote and enhance tumorigenesis in mice (Godinho et al., 2014; Coelho et al., 2015; Serçin et al., 2016; Levine et al., 2017). As centrosome amplification is found in human tumors (Chan, 2011) but not in normal cells, it is an appealing feature to explore for diagnosis, prognosis, and therapy.

Despite being a cancer hallmark, the timing, mechanisms, and impact of centrosome deregulation in human cancer are poorly understood (Godinho and Pellman, 2014). Moreover, whether the incidence of centrosome amplification changes through progression is not known. This partly stems from lack of studies surveying centrosomes at the single-cell level through tumorigenesis. Moreover, most studies score only PCM components, which may not harbor centrioles and thus not represent bona-fide centrosomes (Chan, 2011; Godinho and Pellman, 2014). Understanding the dynamics of centrosome amplification is essential to decipher its role in cancer.

**Figure 1.**
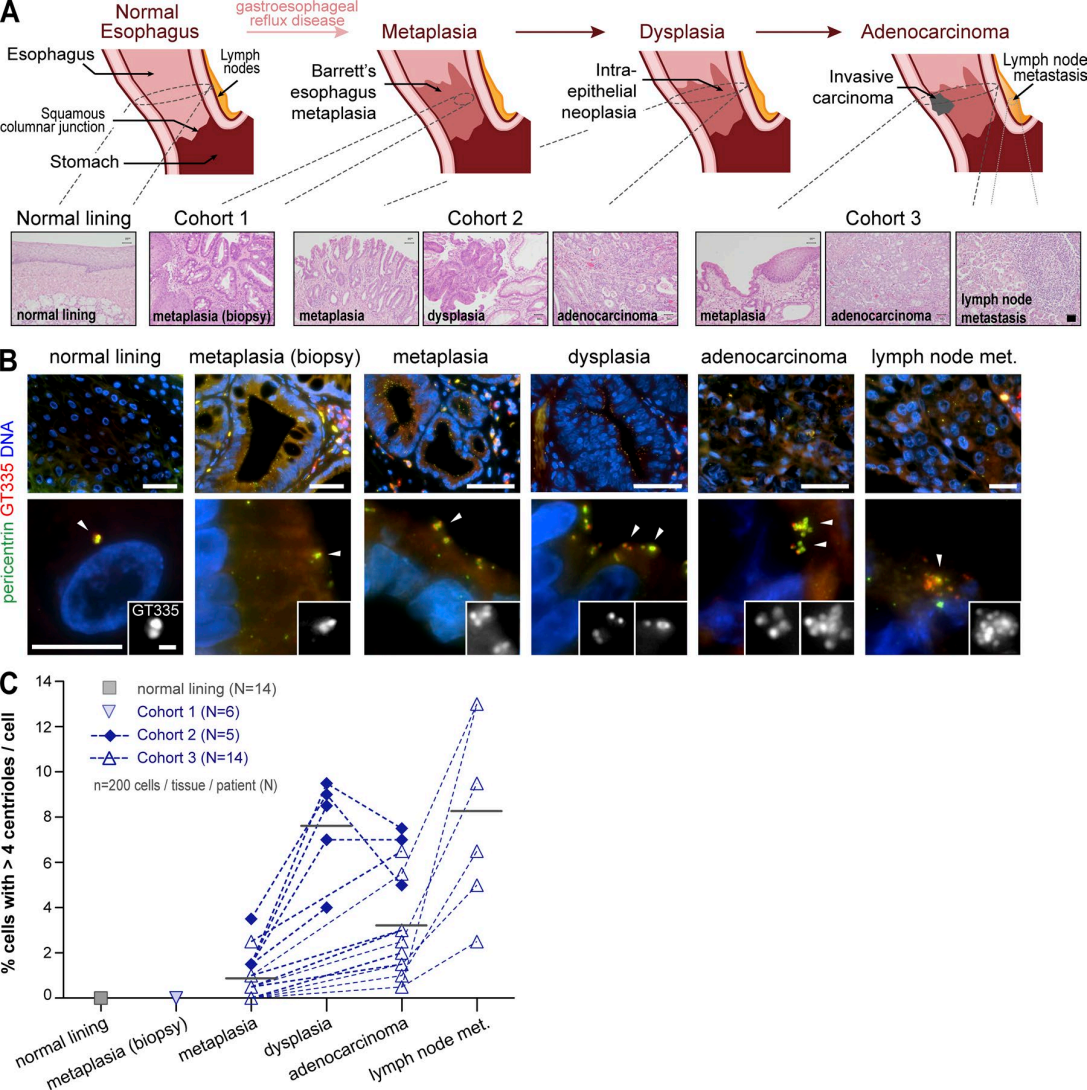
**Centriole amplification arises early and is associated with tumor initiation in patient samples. (A)** BE multistep pathway of progression. Tissue samples’ origins are highlighted. Normal lining: native esophageal epithelium. Cohort 1: metaplasia from biopsies of patients that have not progressed. Cohort 2: dysplasia and adjacent metaplasia as well as foci of adenocarcinoma when present in each patient. Cohort 3: adenocarcinoma and adjacent metaplasia as well as lymph node metastasis (met.) when present in each patient. Representative histopathologic features (H&E) of the samples are shown. Bar, 50 mm. **(B and C)** Samples were stained for PCM (pericentrin), centrioles (GT335), and DNA. **(B)** Representative images with enlargements of cells and centrioles in a single cell (arrowheads). Bars: (top) 50 µm; (bottom, main images) 10 µm; (bottom, insets) 1 µm. **(C)** Quantification of cells with centriole amplification for the tissue samples present in each case analyzed. *n* = 200/tissue/patient. N, number of cases analyzed. Gray lines indicate means of all samples analyzed for each tissue of origin.

It is critical to examine centrosomes along cancer progression. Barrett’s esophagus (BE) is a premalignant condition in which the normal esophageal epithelium is replaced by a stomach/intestine-like metaplastic lining as a result of chronic reflux (Spechler et al., 2011). Its malignant transformation is a multistep process from metaplasia (premalignant condition) to dysplasia (intraepithelial neoplasia), adenocarcinoma (invasive neoplasia), and metastasis (Fig. 1 A; Haggitt, 1994). Given the risk of developing cancer, BE patients are included in a surveillance program (Spechler et al., 2011; Fitzgerald et al., 2014), which allows the study of the intermediate step between normal tissue and tumor initiation. Despite the increasing incidence of esophageal adenocarcinoma, only some BE patients will progress (0.1–0.3%/yr; Hvid-Jensen et al., 2011; Schouten et al., 2011). However, neoplasia resections allow the unique study of sequential stages of progression in each individual patient and thus the more specific detection of consistent differences through progression (Ross-Innes et al., 2015; Stachler et al., 2015).

In this study, we used BE to uncover when and how centrosome amplification arises. We established a method to identify centrosomes at the single-cell level in clinical samples and found that centriole number abnormalities arise early in BE progression both in clinical samples and cell lines. Moreover, we found an increase in abnormalities in dysplasia, which were dependent on p53 loss of function. Our findings suggest centrosome amplification can arise early in human tumorigenesis, being normally repressed by p53.

## Results and discussion

### Centrosome amplification arises as early as the premalignant condition and increases in dysplasia

To determine when centrosome number abnormalities arise, we selected cohorts of patients that allowed us to examine all stages of disease. We therefore included metaplasia samples from biopsies of patients that did not progress (cohort 1) as well as samples from patients subjected to resection upon progression to dysplasia (cohort 2) or adenocarcinoma (cohort 3; Fig. 1 A and Table S1). In these, we analyzed in each patient areas of metaplasia, dysplasia, and adenocarcinoma (cohort 2) along with areas of metaplasia, adenocarcinoma, and lymph node metastasis (cohort 3). As comparison standards for normal epithelial tissue, we examined samples of native esophagus (normal lining; Fig. 1 A) and ileum (Fig. S1 B).

We established a method to identify centrosomes at the single-cell level in tissue samples by immunofluorescence (IF). To ensure centrosome scoring, we labeled its structural components: the centrioles (with glutamylated tubulin) and the PCM (with pericentrin; Fig. 1 B). Thus, only centrioles surrounded by the PCM were scored. Moreover, the background of glutamylated tubulin staining was sufficient to define cell boundaries (Fig. S1 A), thus allowing centriole number scoring cell by cell.

Centriole amplification was never observed in the normal lining of the esophagus (Fig. 1, B and C) or the ileum (Fig. S1, C and D). Although centriole amplification was also not detected in metaplasia from biopsies that had not progressed, cells with supernumerary centrioles were detected early in metaplasia adjacent to dysplasia or adenocarcinoma as well as in all subsequent steps of progression (Fig. 1, B and C; and Fig. S1, C–E). Moreover, the number of centrioles found per cell increased upon progression (Fig. S1 D). Centriole amplification increased significantly from metaplasia to dysplasia (Figs. 1 C and S1 C). Our data also indicate a decrease in adenocarcinoma followed by an increase in metastasis (Figs. 1 C and S1 C). This change in incidence along progression suggests that the percentage of cells with centrosome amplification is dynamic. Our observations suggest that the impact of these abnormalities is likely context dependent, being differently tolerated and having different consequences along progression.

### Loss of p53 function correlates with the increase in centrosome amplification

Mutations in p53, the most mutated gene in human cancers (Petitjean et al., 2007), define the boundary from metaplasia to dysplasia in BE progression (Weaver et al., 2014). As p53 loss is associated with centrosome amplification in many human tumors (Chan, 2011; Godinho and Pellman, 2014), we hypothesized that p53 inactivation is responsible for the increased centrosome amplification observed in dysplasia.

**Figure 2.**
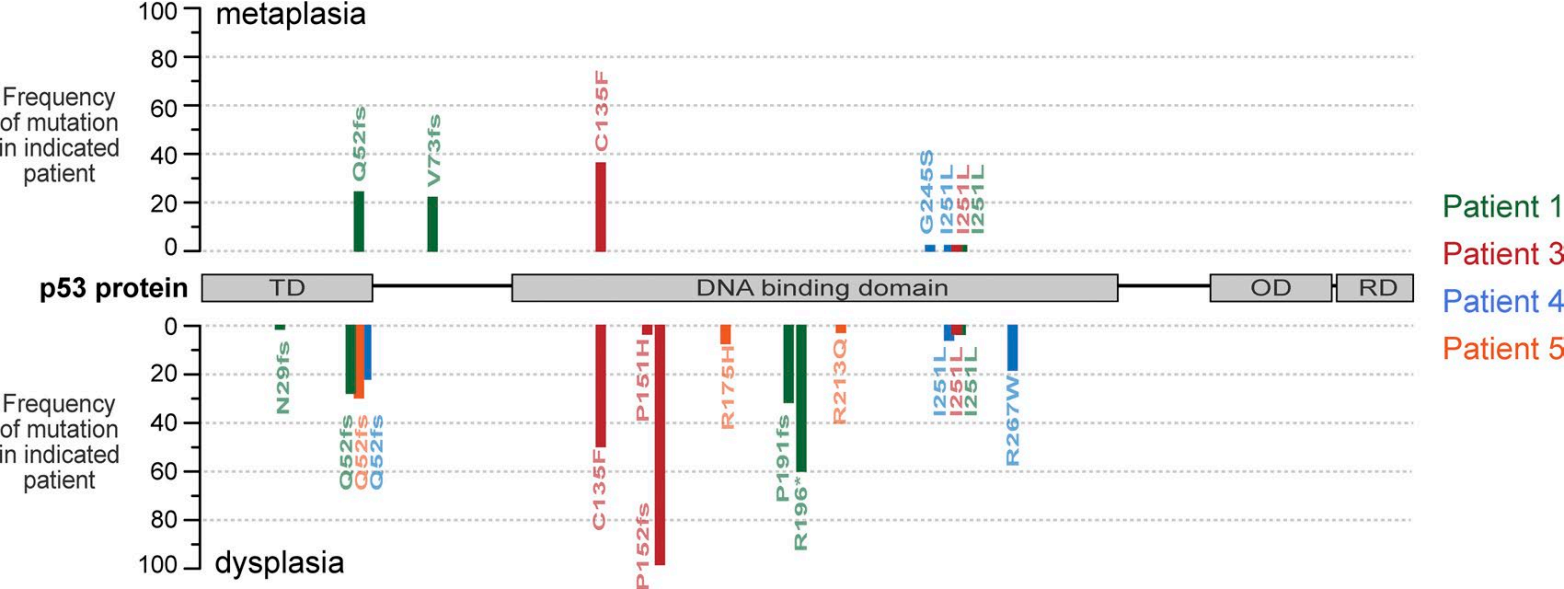
**p53 is deregulated in dysplasia patient samples.** The mutational status of p53 in dysplasia and adjacent metaplasia samples (cohort 2) was determined by NGS. The positions and frequency of the mutations identified in each patient in metaplasia and dysplasia areas are shown above and below the protein schematic, respectively. fs, frameshift mutation; OD, oligomerization domain; RD, regulatory domain; TD, transactivation domain. The asterisk indicates a nonsense mutation.

To test this, we sequenced p53 in metaplasia and dysplasia samples from the same patient (cohort 2). In agreement with previous studies (Hamelin et al., 1994; Gleeson et al., 1995, 1998; Del Portillo et al., 2015), we found that p53 was mutated in dysplasia: all samples contained multiple mutations in high frequency, with some individual mutations being detected in 97% of the reads, whereas metaplasia samples either retained WT p53 or had fewer mutations in lower frequency (Fig. 2 and Table S2). In the BE clinical setting, p53 status is assessed by immunohistochemistry (IHC), a reliable method recommended to aid the dysplasia diagnosis as it detects mutational and nonmutational changes leading to p53 inactivation (Bian et al., 2001; Kaye et al., 2010; Fitzgerald et al., 2014). Using this approach, we confirmed that all dysplasia samples had abnormal p53 expression, indicative of p53 mutations or loss, whereas most metaplasia samples retained WT p53 expression (Fig. S2 and Table S2). Collectively, these results confirm that p53 is first altered in dysplasia and suggest that this change underlies the increased penetrance of centrosome amplification detected at this stage.

### Profile of centrosome amplification in cell lines is similar to patient samples

To test the consequences of p53 loss in centrosome amplification, we took advantage of a well-characterized cell line panel established from all stages of BE progression and containing genomic alterations found in vivo: metaplasia cells are diploid and have WT p53, whereas dysplasia cells are aneuploid and have distinct p53 mutations (Fig. S3 A and Table S3; Palanca-Wessels et al., 2003; Jaiswal et al., 2007). We therefore first asked whether this panel showed a similar trend in centriole amplification along progression to that observed in patient samples. As comparison standards for normal cells, we used native epithelia-derived cells (Table S3; Harada et al., 2003). To assess centrioles, we used two markers (glutamylated tubulin and centrin) in mitotic cells, which normally have four centrioles.

**Figure 3.**
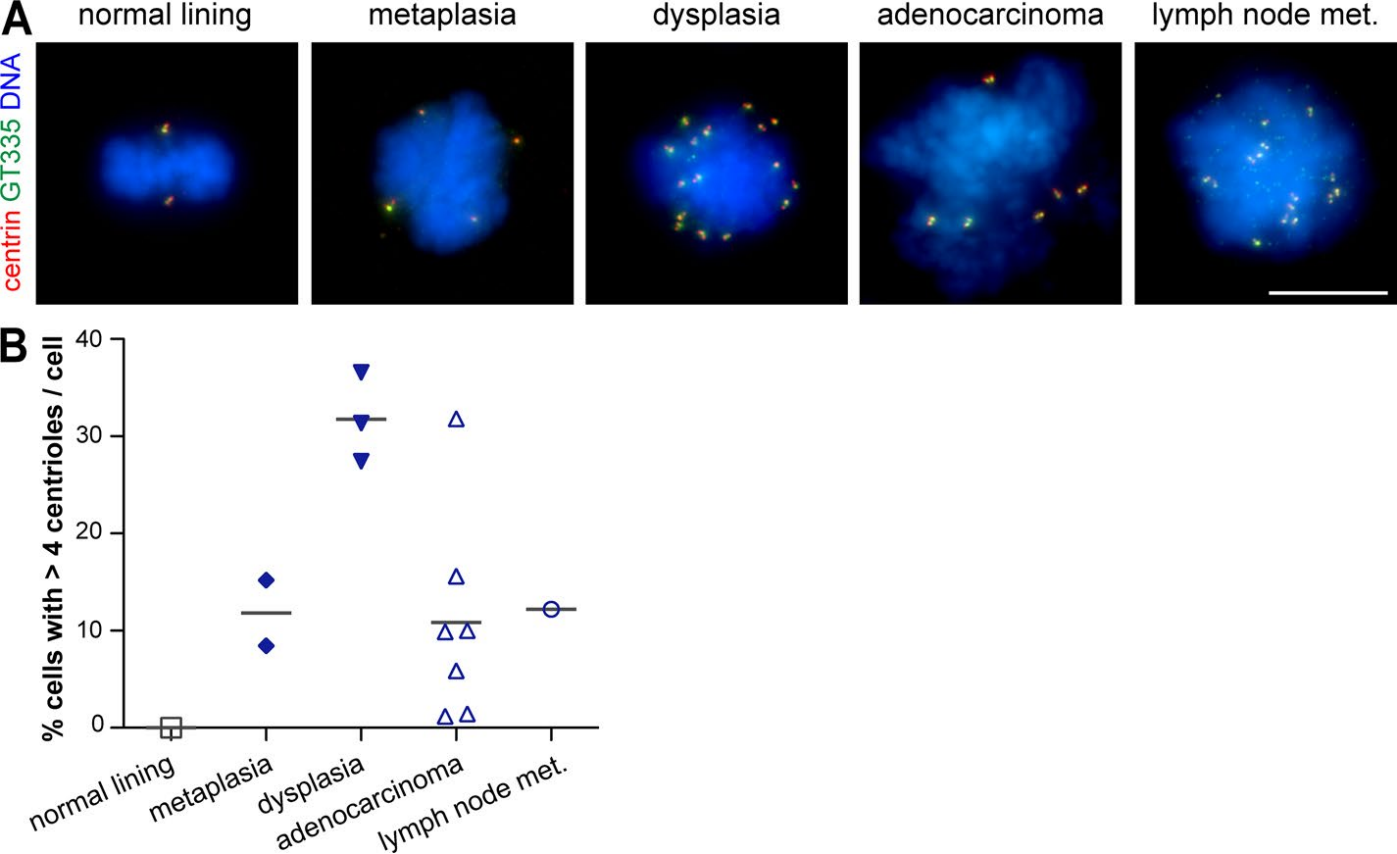
**Profile of centriole amplification in representative cell lines is similar to that in patient samples. (A and B)** Cells derived from the normal lining and from all stages of BE progression were stained for centrioles (centrin and GT335) and DNA. **(A)** Representative images. Bar, 10 µm. **(B)** Quantification of mitotic cells with centriole amplification in each cell line (*n* ≥ 60/cell line) of the indicated tissue of origin. Gray lines indicate means of all cell lines for each tissue of origin. Met., metastasis.

As in tissue samples, centriole amplification was not found in normal lining cells, but it was detected in metaplasia cells and in all cell lines from the subsequent stages (Fig. 3). Moreover, the number of centrioles found per cell increased upon progression (Fig. S3 B). Importantly, the incidence of centriole amplification increased from metaplasia to dysplasia (Fig. 3). This was validated with an additional centriolar marker and confirmed in interphase cells (Fig. S3, C and D). The higher percentage of cells with amplification observed in cell lines compared with tissue samples was likely caused by undercounting in tissue samples, which resulted from technical limitations (see Materials and methods). Interestingly, we had in our collection both an adenocarcinoma cell line (ESO51) and the tumor it was derived from (case 8 in cohort 3), and both had a lower degree of amplification (10% cell line and 2.5% tissue) as compared with the other lines and tumors (up to 31.8% cell lines and 6.5% tissues). Collectively, these observations suggest cell lines keep the centrosome characteristics of their tissue of origin and are thus a good model to test the molecular changes underlying centrosome amplification.

### WT p53 controls centriole amplification in metaplasia

**Figure 4.**
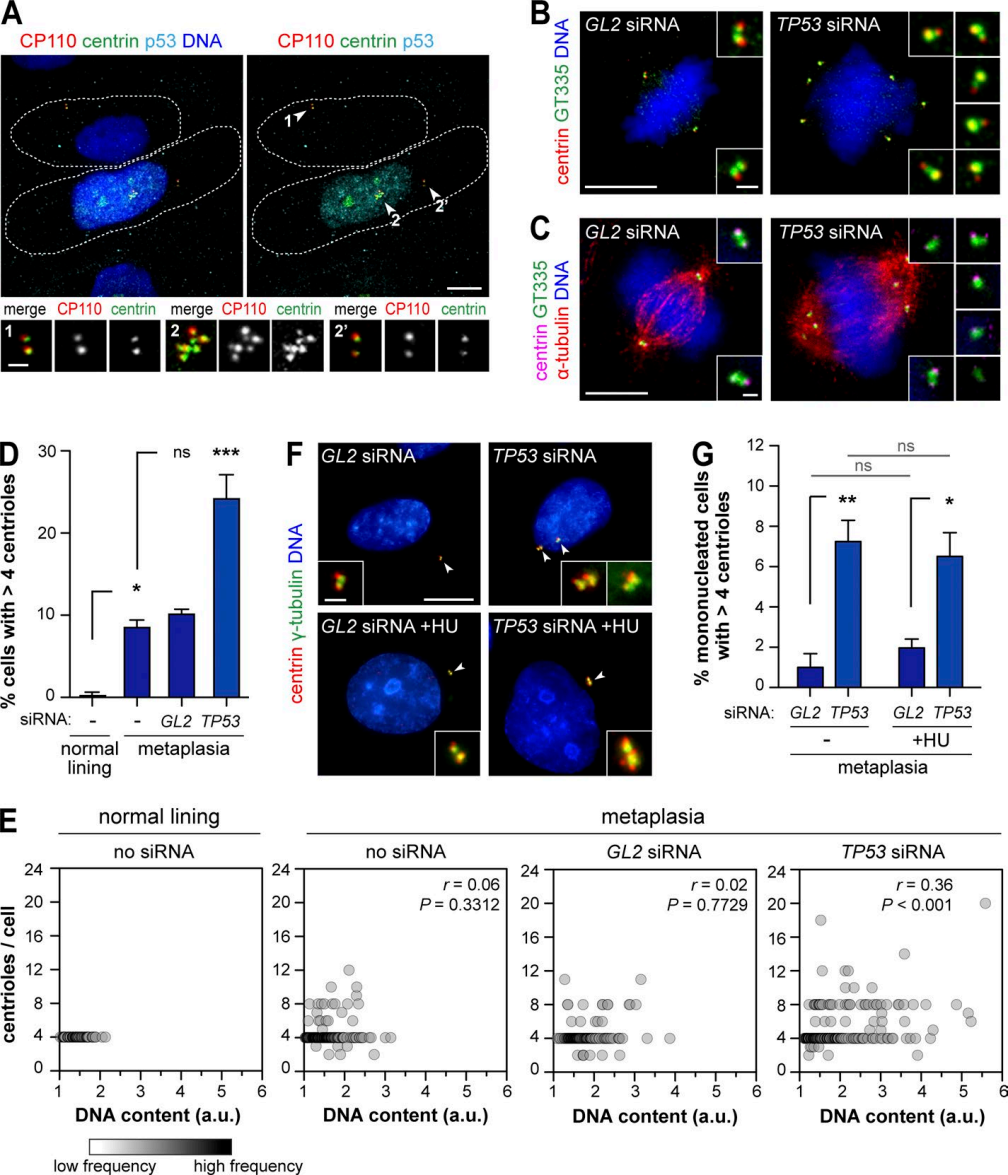
**p53 represses centriole amplification in metaplasia. (A)** Metaplasia cells were stained for p53, centrioles (centrin and CP110), and DNA. Dashed lines denote individual cell outlines given by the CP110/centrin background signal. Insets show centrioles (arrowheads) in p53-negative (1) and p53-positive (2 and 2’) cells. **(B–E)** Metaplasia cells transfected with control (*GL2*) or p53 (*TP53*) siRNA were stained for centrioles (centrin and GT335) and DNA (B) or centrioles, microtubules (α-tubulin), and DNA (C). Untreated metaplasia and normal lining cells were also analyzed. **(B and C)** Representative images with enlargements of centrioles. Bars: (main images) 10 µm; (insets) 1 µm. **(D)** Quantification of mitotic cells with centriole amplification. *n* ≥ 100/condition/experiment. **(E)** Correlation between centriole number and DNA content in each mitotic cell (individual circles). Data are from two independent experiments. *n* ≥ 100/condition/experiment; Spearman test. **(F and G)** Asynchronous (−) or S phase–arrested (hydroxyurea [+HU]) metaplasia cells transfected with control (*GL2*) or p53 (*TP53*) siRNA were stained for centrioles (centrin) and γ-tubulin. **(F)** Representative images with enlargements of centrioles (arrowheads). Bars: (main images) 10 µm; (insets) 1 µm. **(G)** Quantification of cells with centriole amplification. *n* ≥ 60/condition/experiment. Error bars show means ± SEM of three independent experiments. *, P < 0.05; **, P < 0.01; ***, P < 0.001 (ANOVA).

Previous work showed that p53 loss alone in normal human cells does not lead to centrosome number defects. However, loss of p53 is required for the survival of cells experimentally perturbed to gain or lose centrosomes (Cuomo et al., 2008; Holland et al., 2012; Lambrus et al., 2015; Wong et al., 2015). Given the small population of cells with supernumerary centrioles in metaplasia, we hypothesized that there is underlying centrosome amplification in metaplasia that is normally suppressed by p53. Cellular stress normally induces p53, leading to its nuclear accumulation and activation of downstream effectors to prevent the expansion of those cells (Rivlin et al., 2011). We found that all interphase metaplasia cells with centriole amplification showed p53 nuclear accumulation, whereas the majority (70%) of cells with normal centriole number had undetectable p53 (Fig. 4 A). To test whether p53 was preventing the expansion of cells with amplification, we depleted p53 by siRNA (Fig. S3 E). Indeed, p53 depletion in metaplasia resulted in an increase in centriole amplification to similar levels detected in dysplasia (Fig. 4, B–D). This result was confirmed using different p53 siRNAs or shRNA (Fig. S3, F–I). Significantly, p53 depletion alone was not sufficient to generate centriole amplification in normal lining cells (Fig. S3, J and K). It is therefore likely that yet-unidentified molecular changes occurring in metaplasia (Weaver et al., 2014) promote centriole amplification at this stage.

Importantly, supernumerary centrioles in metaplasia both before and after p53 loss were active, as they were able to recruit γ-tubulin and nucleate microtubules (Figs. 4 C and S3, L and M), thus potentially contributing to genomic instability (Ganem et al., 2009; Silkworth et al., 2009). Future studies are needed to elucidate the fate of metaplasia cells dividing with supernumerary centrioles. In the absence of p53, an increase in centrosome amplification may play a role in tumor initiation by conferring the genomic instability required for the acquisition of malignant properties. In agreement with this, chromosomal instability was detected in metaplasia adjacent to neoplasia and was progressively more frequent in dysplasia and adenocarcinoma (Chaves et al., 2007; Paulson et al., 2009).

Centrosome number deregulation can occur by several mechanisms including centrosome biogenesis deregulation and cytokinesis failure (Godinho and Pellman, 2014). In the latter, centrosome numbers increase in concert with ploidy (Davoli and de Lange, 2011). Ploidy is known to be deregulated in BE tumorigenesis: tetraploidy was detected in BE and predicts progression to aneuploidy, which is preceded by p53 changes (Reid et al., 2010). Moreover, ploidy deregulation is likely also surveyed by p53 (Thompson and Compton, 2010; Ganem et al., 2014). To test the association between deregulation of ploidy and centriole numbers, we investigated both features in metaplasia cells with or without p53. We detected ploidy deregulation in mitotic metaplasia cells (Fig. 4 E) and binucleated cells in metaplasia that elicited a p53 response (Fig. S3 N). Ploidy deregulation was aggravated upon p53 silencing (Fig. S3, O–Q). Moreover, both centriole number and ploidy increased upon p53 loss (Fig. 4 E), suggesting a common origin such as cell division failure. If centriole amplification detected upon p53 loss in metaplasia results exclusively from cell division failure, then blocking metaplasia cells in S phase and thus not allowing them to divide should abrogate the increase in amplification. We found that p53 loss was still able to promote centriole amplification in S phase–arrested metaplasia cells (hydroxyurea treatment; Fig. 4, F and G; and Fig. S3, R and S), suggesting that at least part of the amplification observed does not result from failed cell division. Previous work showed that S phase arrest was sufficient to generate centrosome amplification in p16-deficient human mammary cells (McDermott et al., 2006). However, this was not the case in control metaplasia cells (Fig. 4, F and G), which also lack the tumor suppressor p16, one of the earliest changes in BE (Table S3; Reid et al., 2010). The contribution of this and other early events to centrosome amplification deserves further study. Collectively, these results suggest centriole amplification can arise independently of cell division failure in BE metaplasia and demonstrate a key role for p53 in preventing the expansion of those cells.

### p53 hotspot mutations R175H and R248W deregulate centriole number control

**Figure 5.**
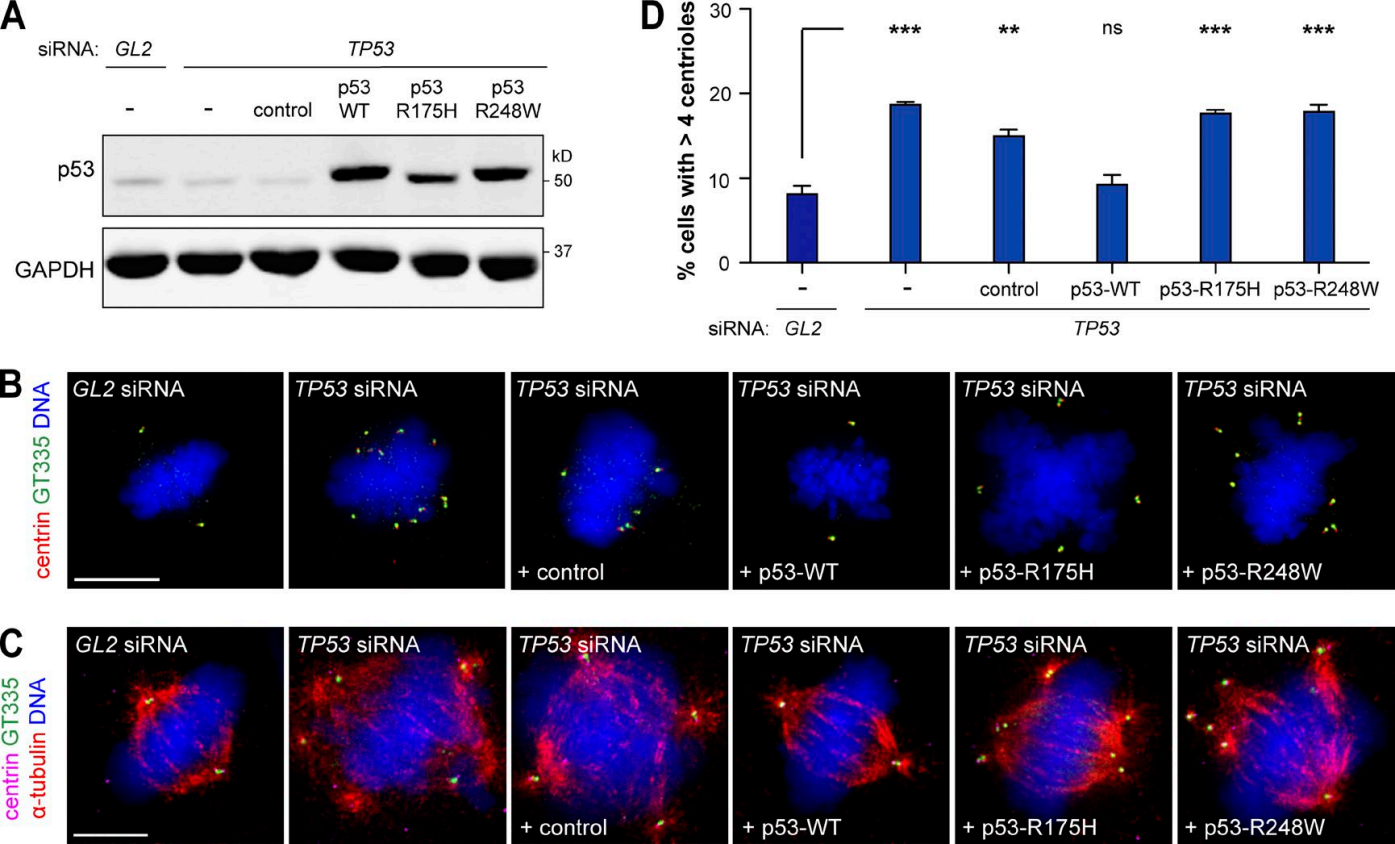
**The p53 hotspot mutations R175H and R248W deregulate centriole numbers in metaplasia. (A–D)** Metaplasia cells depleted of endogenous p53 (*TP53*) were transfected with WT p53, p53-R175H, p53-R248W, or the empty plasmid (control). Metaplasia cells transfected with control siRNA (*GL2*) or siRNA against endogenous p53 (*TP53*) alone were also analyzed. **(A)** Protein levels were assessed by WB. GAPDH was used as a loading control. **(B–D)** Cells were stained for centrioles (centrin and GT335) and DNA (B) or centrioles, microtubules (α-tubulin), and DNA (C). **(B and C)** Representative images are shown. Bars, 10 µm. **(D)** Quantification of mitotic cells with centriole amplification. *n* ≥ 100/condition/experiment. Error bars show means ± SEM of two independent experiments. **, P < 0.01; ***, P < 0.001 (ANOVA).

As most tumor suppressors, p53 inactivation can be caused by nonsense or frameshift mutations that lead to a truncated nonfunctional protein. In most cases, however, including BE tumorigenesis, p53 contains a missense mutation resulting in the expression of a full-length protein that loses the WT function and may gain oncogenic function (Fig. 2; Rivlin et al., 2011; Weaver et al., 2014). Hence, it is relevant to study the effect of p53 missense mutations on centrosome number as it could be different from loss of WT function. Notably, all three dysplasia cell lines, which have either a frameshift mutation or the missense mutations R175H or R248W, exhibited similar levels of centriole amplification (Fig. 3 B and Table S3). R175H and R248W are known hotspot mutations in BE neoplasia and other tumors (Fig. 2; Petitjean et al., 2007; Weaver et al., 2014). Expression of these mutants in p53^−/−^ MEFs and in a lung metastasis cell line led to centrosome amplification (Tarapore et al., 2001; Noll et al., 2012). In this study, we tested whether expression of R175H or R248W mutants prevents the amplification elicited by p53 loss in metaplasia (Fig. 5 A). In contrast with expression of WT p53, neither mutant prevented the accumulation of cells with amplification (Fig. 5, B–D). Moreover, amplified centrioles were active as they nucleated microtubules (Fig. 5 C). These results show both residues are essential for p53 to control centriole number in metaplasia and that loss of WT p53 function leads to increase in centriole amplification upon progression from metaplasia to dysplasia. Moreover, previous findings that R175H and R248W mutations can promote genomic instability and invasion (Rivlin et al., 2011; Muller and Vousden, 2014) further support a role for centrosome amplification in those processes. Further studies are needed to determine how distinct p53 mutations affect tumorigenesis and whether that is related to centrosome amplification.

In summary, we showed that centrosome amplification (A) is never observed in native epithelia, suggesting centriole number control is robust in the normal population; (B) it arises as early as the premalignant condition and is present in all stages in all patients; (C) its incidence is dynamic during progression; (D) it significantly increases from metaplasia to dysplasia; and (E) this increase correlates with and is dependent on loss of p53 function. These findings have important implications in our understanding of centrosome amplification in cancer progression.

An association between p53 loss and centrosome amplification is found in several cancers (Chan, 2011). Our findings clarify this relationship in human cancer: centrosome amplification, though low in incidence, arises in the context of functional WT p53 that plays a crucial role in preventing widespread centrosome amplification. Our study thus supports the existence of a p53-dependent pathway preventing proliferation upon centrosome number deregulation (Ganem et al., 2014; Lambrus et al., 2015; Fava et al., 2017). Future work will be important to elucidate the mechanisms activating p53 upon centrosome amplification.

Our analysis at the single-cell level also revealed that despite the dynamic clonal evolution in BE progression (Reid et al., 2010; Weaver et al., 2014), centrosome amplification is never eliminated nor close to 100%. As centrosome amplification leads to genomic instability (Ganem et al., 2009; Silkworth et al., 2009), ability to invade (Godinho et al., 2014; Kushner et al., 2014), and non–cell-autonomous effects (Marusyk et al., 2014; Ganier et al., 2018), it does not have to be present in a high fraction of cells to impact tumor progression. This suggests cells with centrosome deregulation may be advantageous at the population level by promoting the fitness of the other cells.

Given widespread occurrence of p53 mutations and centrosome amplification in human tumors, our findings on the timing and ordering of these events and aneuploidy in BE tumorigenesis are likely to be extended to other cancers. Moreover, the clarification of the relationship between centrosome amplification and loss of p53 function suggests that this can be part of a wanting gene signature that predicts significant centrosome amplification in tumor samples. This could be useful to identify patients that will respond to centrosome-related inhibitors currently in clinical trials (Godinho and Pellman, 2014; Mason et al., 2014). Finally, the cell lines used in this study will be an excellent tool to get further insight into how supernumerary centrosomes arise and how they contribute to tumor progression, invasiveness, and metastasis.

## Materials and methods

### Patient selection and clinical samples

For the purpose of this study, three cohorts of patients were selected from the Pathology Department database of Instituto Português de Oncologia de Lisboa Francisco Gentil (IPOLFG). Cohort 1: biopsies from six BE patients included in the surveillance program, with metaplasia negative for dysplasia until the moment of this study (in a followup from 1998–2012). An additional set of similar biopsies from another 22 BE patients was evaluated separately (Fig. S1 E). Cohort 2: five patients included in the surveillance program that were submitted to endoscopic resection or esophagectomy upon progression to high-grade dysplasia or adenocarcinoma. Cohort 3: 14 patients that when first examined already had adenocarcinoma and were submitted to esophagectomy (without neoadjuvant therapy). All formalin-fixed paraffin-embedded (FFPE) samples were selected not compromising future diagnostic studies. Areas of metaplasia, dysplasia, adenocarcinoma, and/or lymph node metastasis were selected. All cases were anonymized after a clinical record review for demographic data. Staging and grading were performed according to the American Joint Committee on Cancer staging system and the World Health Organization criteria, respectively (Amin et al., 2010; Bosman et al., 2010). As standards of comparison for normal squamous- and columnar-lined mucosa, respectively, the squamous-lined mucosa from the proximal margin of 14 total gastrectomies for gastric adenocarcinoma and the ileal mucosa from the proximal margin of 14 right-hemicolectomies for intestinal adenocarcinoma were used. Material from BE patients was obtained in the context of IPOLFG surveillance program and was used without compromising future patient management. All samples were routinely anonymized upon collection for the archival file, thus guaranteeing the privacy, confidentiality, and protection of patients and their personal data. This study was approved by the IPOLFG Research Council and Ethics committee.

### Cell culture

Human telomerase-immortalized (hTERT) BAR-T and BAR-T10 cell lines derived from biopsies of patients with nondysplastic BE as well as BAR-T cell lines expressing the pSUPER-p53RNAi or the control empty vector pSUPER-retro.neo (all from R. Souza, Baylor University Medical Center, Dallas, TX; Jaiswal et al., 2007; Zhang et al., 2010) were cocultured with a fibroblast feeder layer (Swiss 3T3 cells [85022108; European Collection of Authenticated Cell Cultures] treated with 10 µg/ml mitomycin C [Sigma-Aldrich] for 2 h) and maintained in KBM2 medium (Lonza) supplemented with 5% FBS, 0.1 nM cholera toxin (Sigma-Aldrich), 70 µg/ml bovine pituitary extract (BPE; Sigma-Aldrich), 400 ng/ml hydrocortisone (Sigma-Aldrich), 20 ng/ml EGF, 20 µg/ml adenine (Sigma-Aldrich), 5 µg/ml insulin (Sigma-Aldrich), 5 µg/ml transferrin (Gibco), and 100 U/ml penicillin-streptomycin (Gibco). Cells were seeded in wells precoated with collagen IV (1 µg/cm^2^; Sigma-Aldrich) for individual experiments. hTERT CP-B, CP-C, and CP-D cell lines derived from biopsies of patients with high-grade dysplasia (from P. Rabinovitch, University of Washington, Seattle, WA; Palanca-Wessels et al., 2003) were maintained in MCDB 153 medium (Sigma-Aldrich) supplemented with 5% FBS, 0.4 µg/ml hydrocortisone, 20 ng/ml EGF, 1 nM cholera toxin, 140 µg/ml BPE, 20 µg/ml adenine, 4 mM glutamine (Gibco), 0.1% insulin-transferrin-sodium selenite (Sigma-Aldrich), and 100 U/ml penicillin-streptomycin. Adenocarcinoma-derived cell lines ESO26, ESO51 (both established previously; Boonstra et al., 2010), OE19, OE33, FLO-1, SK-GT-4, OACP4, KYAE-1, and lymph node metastasis-derived cell line OACM5.1 (from W. Dinjens, Erasmus Medical Center Cancer Institute, Rotterdam, Netherlands) were grown in RPMI 1640 or in RPMI 1640–Ham’s F12 (Kyae-1 cell line) supplemented with 10% FBS and 100 U/ml penicillin-streptomycin. The ESO51 cell line was derived from the tumor in case 8 from cohort 3 (Table S1). All cell lines have been recently validated (Boonstra et al., 2010). As standards of comparison for normal cells, we used hTERT normal esophageal epithelial cells (EPC2; from S. Godinho, Barts Cancer Institute, London, England, UK) as well as a common experimentally used nontransformed hTERT cell line derived from normal human RPE1. hTERT-ECP2 was grown in keratinocyte-serum-free medium with glutamine supplemented with EGF, BPE (Gibco), and 100 U/ml penicillin-streptomycin (Harada et al., 2003), and hTERT-RPE1 cells were grown in DMEM-F12 (Gibco) supplemented with sodium bicarbonate, 10% FBS, and 100 U/ml penicillin-streptomycin. All cells were grown at 37°C in a 5% CO_2_ atmosphere and tested for the presence of mycoplasma.

### IF microscopy

#### Tissue samples

From each FFPE block, 3-µm-thick tissue sections were transferred to positively charged glass slides and oven dried (70°C) for at least 1 h. Sections were then deparaffinized in xylene, placed in 100% ethanol, treated with 2% hydrogen peroxide in methanol solution for 10 min to block the endogenous peroxidase, and then washed in distilled water. Antigen retrieval was done in a pressure cooker in a 0.01-M sodium citrate–buffered solution, pH 6, for 6 min followed by incubation with a blocking buffer (TBS with 5% BSA) for 10 min at RT. Slides were then incubated with primary antibodies diluted in Bond primary antibody diluent (Leica Microsystems) with background-reducing components for 1 h at RT followed by washes in TBS before incubation with secondary antibodies for 30 min at 37°C. Slides were then washed extensively with TBS, dehydrated through gradient alcohols, and mounted in Vectashield with DAPI for DNA staining (Vector Laboratories). 

#### Cell lines

Cells were grown on coverslips and were fixed with ice-cold methanol at −20°C for 10 min. Standard IF procedures involved blocking (30 min) and antibody incubations (overnight at 4°C for 1 h at RT) in PBS with 10% FBS, and washes were performed in PBS. Coverslips were mounted on glass slides in Vectashield with DAPI (Vector Laboratories). For DNA content analysis by IF, coverslips were incubated for 10 min in PBS with 1 µg/ml Hoechst 33342 (Invitrogen) before being mounted in Vectashield (Vector Laboratories). 

#### Image acquisition

Images were obtained at RT using a T*i*-E inverted microscope (Nikon) with a Plan Apochromat VC 100× 1.40 NA oil objective, an ORCA ER2 charge-coupled device camera (Hamamatsu Photonics), and Nikon software or with an Eclipse T*i*-E (Nikon) microscope with a Plan Apochromat 100× 1.49 NA oil objective, an Evolve electron-multiplying charge-coupled device camera (Photometrics), and MetaMorph software (Molecular Devices). Images were acquired as a z series (0.2-µm z interval) and are presented as maximum-intensity projections. Images were prepared using Photoshop (Adobe) and ImageJ (National Institutes of Health). 

#### Antibodies

Primary antibodies used were against glutamylated tubulin (1:800 [tissue sections] and 1:500 [cell lines]; mouse; GT335; AdipoGen), pericentrin (1:250; rabbit; ab4448; Abcam), centrin (1:500; rabbit; N-17; Santa Cruz Biotechnology, Inc.), centrin (1:500; mouse; 20H5; EMD Millipore), A647-conjugated centrin (1:500; mouse; 20H5; EMD Millipore), γ-tubulin (1:500; mouse; GTU88; Sigma-Aldrich), α-tubulin (1:50; rat; YL1/2; AbD Serotec), p53 (1:100; mouse; DO-1; EMD Millipore), E-cadherin (1:30; rabbit; Cell Signaling Technology), and CP110 (1:250; rabbit; Jiang et al., 2012). The secondary antibodies FITC, Cy5, and rhodamine red (1:50 [tissue sections] and 1:200 [cell lines]; Jackson ImmunoResearch Laboratories, Inc.) as well as Alexa Fluor 488 and 647 (1:500 [cell lines]; Thermo Fisher Scientific) were also used.

### Centrosome/centriole number and DNA content analysis by IF

#### Tissue samples

We used two markers that have robust staining in paraffin-embedded samples and that label the two structural components of the centrosome: the centrioles (marked by glutamylated tubulin [GT335]) and the PCM (marked by pericentrin). Centriole number was assessed by GT335 when it colocalized with pericentrin, thus identifying centrosomes. To achieve good staining and resolution at the cellular level, we used thin tissue samples (3 µm thick, as normally used in the clinic). Immunostaining of centrosomes was judged satisfactory when it was detected in the adjacent epithelium. The analysis was done taking into consideration the limitations derived from the specificities of working in FFPE samples: cellular truncation and cell overlapping. We first tested our accuracy of counting centrioles using GT335 marker with or without a costaining with a membrane marker (E-cadherin) and found that the background of GT335 staining was sufficient to distinguish cell limits and that the results obtained were similar. The counts were performed by going through all the z series acquired (see IF microscopy section) covering the whole depth of the section to assess the transversal and sagittal plans of each cell, thus identifying the transition to adjacent cells. Cells whose limits could not be clearly distinguished as well as cells overlapping with neighboring cells were not considered. To test for the occurrence of undercounting and overcounting related to the usage of histological sections, we did an extensive analysis in our standards of comparison for normal cells (14 ileum and 14 squamous) that were cut at the same thickness as all the other samples. Given that this analysis was performed in typically well-differentiated areas (i.e., not proliferative), the number of centrosomes expected per cell was one (with two centrioles). Whereas we detected an expected undercounting (cells with 0 centrioles), we never detected an overcounting (cells with more than two centrioles), suggesting that the method used to distinguish cell limits was robust. In each case, at least 200 countable cells with centrosomes were examined. Depending on the cell cycle stage, a cell either has one centrosome with two centrioles (G1) or two centrosomes with two centrioles each (S, G2, and M; Bettencourt-Dias et al., 2011). Because we did not use a cell cycle stage marker, only cells with more than four centrioles were considered to contain an abnormal centriole number content (centriole amplification).

#### Cell lines

Centrioles were considered when paired signals of two centriole markers (GT335, centrin, or CP110) were observed or when a centriole marker (centrin) colocalized with a PCM marker (γ-tubulin). For cell-by-cell centriole number and DNA content analysis in mitotic cells, sum projections of DNA staining (Hoechst) were used to determine total intensity of signal as measured by ImageJ software (National Institutes of Health). Total intensity was corrected according to the background intensity signal: corrected total cell fluorescence = integrated density – (area of selected cell × mean fluorescence of background readings). The number of cells and samples analyzed, number of experiments performed, and statistical analyses are detailed in the figure legends.

### DNA extraction and p53 next-generation sequencing (NGS)

FFPE tissue sections (5 µm) were deparaffinized and counterstained with H&E. Metaplasia- and dysplasia-enriched areas were microdissected with a needle under pathologist’s guidance. Total DNA was extracted with GeneRead DNA FFPE kit (QIAGEN) according to the manufacturer’s instructions with a slight modification: proteinase K cell lysis at 56°C was performed overnight. DNA was eluted in 20 µl of elution buffer. To evaluate DNA concentration and integrity, DNA isolated from each sample was quantified in TapeStation 2200 using the Genomic DNA ScreenTape (Agilent Technologies). Because of the small amounts of extracted DNA from each sample area, DNA was precipitated according to the sodium acetate precipitation of small nuclei acids protocol (Thermo Fisher Scientific). Genomic DNA libraries were prepared using the Ion Ampliseq Library kit (2.0) as well as the community panel Ion Ampliseq TP53 and quantified by quantitative PCR with the Ion Library Quantification kit (Thermo Fisher Scientific). The emulsion PCR of amplified libraries was performed using Ion Chef (Thermo Fisher Scientific). Sequencing runs were performed with Ion personal machine using 316 Chips (Thermo Fisher Scientific) aiming for a mean sequencing depth coverage of 500×. With the exception of one sample where the amount of DNA was too low for robust analysis, we were able to sequence all coding exons of p53 by NGS in all paired samples.

### Analysis of p53 by IHC

Analysis was performed as currently used to assess p53 in morphological lesions using tissue samples. Staining of p53 (1:150; mouse; DO-7; Cell Marque) was performed on either a fully automated IHC BOND III system (Leica Biosystems) using ER1 solution (15 min) for antigen retrieval and Novocastra bond polymer refine detection or on a Ventana Benchmark Ultra (Roche) using the CC1 solution (24 min) for antigen retrieval and an OptiView DAB IHC detection kit (Ventana). An expert pathologist qualitatively evaluated p53 immunostaining as WT, positive, or negative. WT expression: weak positivity similar to the observed in the normal native epithelium (internal control). Positive expression: the intensity of staining was graded as weak, moderate, or intense compared with the native epithelium and as focal or diffuse according to the amount of positive cells (<10% vs. >10%). Negative expression: complete absence or only occasional scattered positive cells within a context of WT staining (metaplasia or native epithelium).

### RNAi, transfection, and drug treatment

Endogenous *TP53* was depleted using siRNA oligonucleotides (5′-GGGAGTTGTCAAGTCTCTG-3′; Sigma-Aldrich) targeting the 3′ UTR region. The *TP53* gene was alternatively depleted using other siRNA oligonucleotides: *TP53^1^* (sc-29435; Santa Cruz Biotechnology, Inc.) or *TP53^2^* (5′-GACTCCAGTGGTAATCTAC-3′; Sigma-Aldrich; same sequence as in BAR-T–pSUPER-p53RNAi cell line [Brummelkamp et al., 2002]). Luciferase (*GL2*) siRNA (5′-CGTACGCGGAATACTTCGA-3′; Takara Bio Inc.) was used as a control. Cells were transfected with 50 nM siRNA for 72 h using Lipofectamine RNAiMAX (Thermo Fisher Scientific). For S phase arrest, 16 h after siRNA treatment, cells were treated with 4 mM hydroxyurea (Sigma-Aldrich) for 48 h. Transient plasmid transfections were performed with Lipofectamine LTX (Invitrogen) according to the manufacturer’s instructions 16 h after siRNA transfection and were analyzed after 48 h. p53 constructs were obtained from Addgene (Baker et al., 1990).

### Cell lysis, SDS-PAGE, and Western blotting (WB)

Cells were harvested and pelleted before snap freezing in liquid nitrogen. Cell lysates were prepared by resuspending pellets in lysis buffer (50 mM Hepes, pH 7.4, 100 mM KCl, 1 mM EGTA, 1 mM MgCl_2_, 10% glycerol, 0.05% NP-40, 1× protease inhibitor cocktail, and 1× phosphatase inhibitor cocktail) for 10 min on ice. Lysates were then centrifuged for 10 min at 14,000 rpm at 4°C, and protein concentration of the cleared supernatant was determined by Bradford assay. Laemmli buffer was added to the samples to 1× and then boiled at 99°C for 5 min before analysis on polyacrylamide gels. Standard WB procedures involved blocking in TBS supplemented with 5% milk powder and 1% milk powder in TBS-T (0.1% Tween-20 in TBS) for antibody incubations, and washes were performed in TBS-T. Primary antibodies were against p53 (1:1,000; mouse) and GAPDH (1:1,000; rabbit; 14C10; Cell Signaling Technology). IRDye secondary antibodies were used at 1:10,000 and were purchased from Odyssey and LI-COR Biosciences.

### Cell cycle phase distribution and DNA content analysis by flow cytometry

Cells were harvested, pelleted, and washed in 1× PBS before being fixed in 70% ice-cold ethanol and kept on ice for 30 min. After washes with 1× PBS, cells were resuspended in 1× PBS with 100 µg/ml RNase A (QIAGEN) and 100 µg/ml propidium iodide (Sigma-Aldrich) and incubated at 37°C for 30 min in the dark. Cells were then analyzed with FACScan (BD).

### Online supplemental material

Fig. S1 shows analysis of centriole numbers in patient samples. Fig. S2 shows analysis of p53 status in patient samples by IHC. Fig. S3 shows centriole number and ploidy in cell lines. Table S1 shows centriole number analysis in paraffin-embedded tissue. Table S2 shows analysis of p53 status. Table S3 shows cell line information and centriole analysis.

## Supplementary Material

Supplemental Materials (PDF)

Table S3 (Excel)
