# Supplementary material for: Centrosome amplification arises before neoplasia and increases upon p53 loss in tumorigenesis
Source: J Cell Biol. 2018 Jul 2;217(7):2353–63. doi: 10.1083/jcb.201711191 (PMC6028540; doi:10.1083/jcb.201711191)
Supplement: Supplemental Materials (PDF) [file JCB_201711191_sm.pdf]

Supplemental material

Lopes et al., https://doi.org/10.1083/jcb.201711191

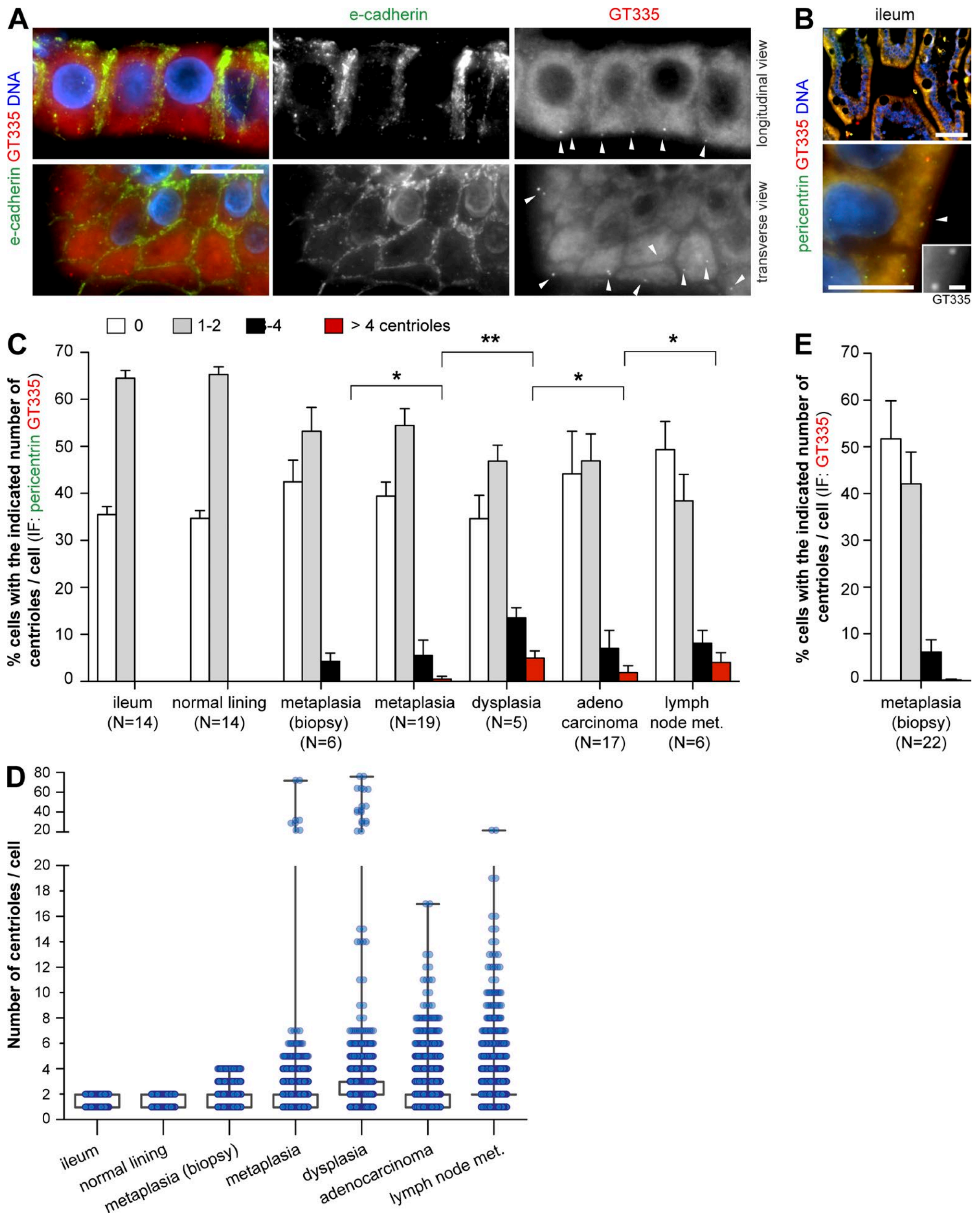

Figure S1. **Analysis of centriole numbers in patient samples. (A and B)** Tissue samples of the normal ileum were stained for the cell membrane (E-cadherin), centrioles (GT335), and DNA (A) or for PCM (pericentrin), centrioles (GT335), and DNA (B). **(A)** Representative images show that the cell's limits, defined by E-cadherin staining, are easily distinguished using solely the GT335 background staining. Arrowheads indicate centrioles. Bar, 10 µm. **(B)** Representative images with enlargements of cells and centrioles in a single cell (arrowhead). Bars: (top) 50 µm; (bottom, main image) 10 µm; (bottom, inset) 1 µm. **(C and D)** The number of centrioles in each cell was analyzed in samples of the normal ileum, of the normal lining of the esophagus, and of the different stages of BE multistep tumorigenesis progression: metaplasia from biopsies of patients that have not progressed to this date (cohort 1) and areas of metaplasia, dysplasia, adenocarcinoma, and lymph node metastasis (met.) from cohorts 2 and 3. Samples were stained for PCM (pericentrin), centrioles (GT335), and DNA. **(C)** Quantification of cells with the indicated centriole number content for the tissue samples present in each case analyzed. $n \geq 200$ cells/tissue/patient. N, number of cases analyzed. Cells with more than four centrioles: *, $P < 0.05$; **, $P < 0.01$ (two-sample Wilcoxon rank-sum [Mann-Whitney]; independent samples; two sided; p-values were adjusted for multiple testing using the Benjamini and Hochberg method). **(D)** Number of centrioles per cell (individual circles) for the tissue samples present in each case analyzed. $n = 200$ cells/tissue/patient; number of cases was analyzed as in C. Individual data points are plotted over the box plots. **(E)** The number of centrioles in each cell was analyzed in metaplasia samples from biopsies of another 22 patients that have not progressed to this date. Samples were stained for centrioles (GT335) only and DNA. Quantification of cells with the indicated centriole number content. $n \geq 200$ cells/patient. N, number of cases analyzed. Error bars show means ± SD.

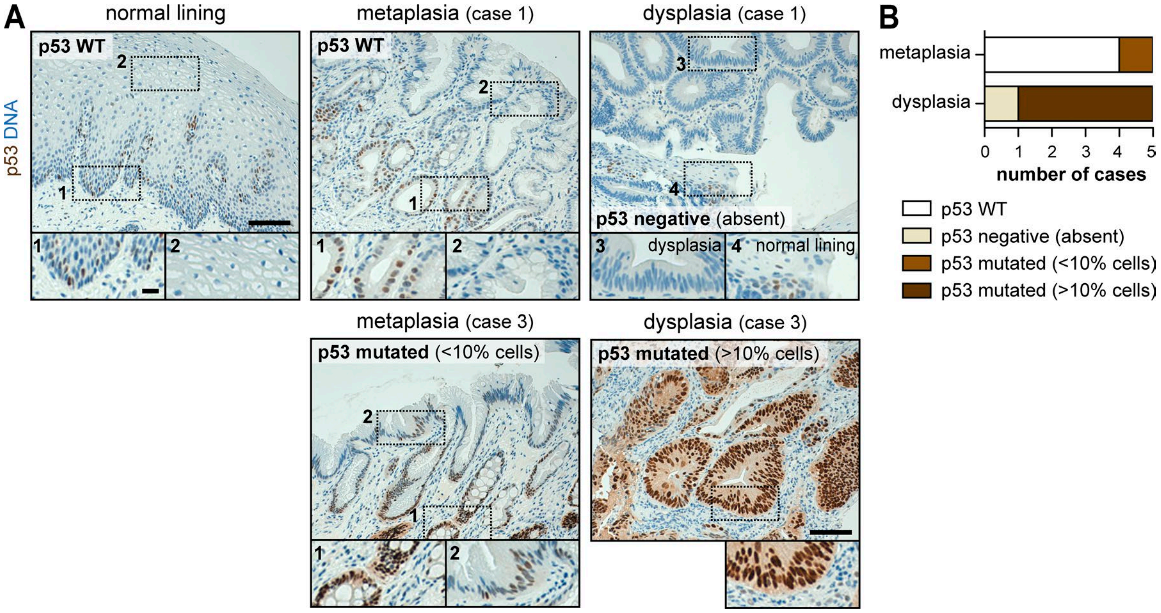

Figure S2. **Analysis of p53 status in patient samples by IHC. (A and B)** p53 expression was analyzed by IHC in tissue samples of the normal lining and in metaplasia and dysplasia samples from the same patient (cohort 2). **(A)** Representative images of p53 protein expression in the different tissue samples. DNA is shown in blue. Insets show 2× magnification of indicated areas. As WT p53 protein has a short half-life, it is only weakly detected in the nucleus of some proliferating cells (1) and not in differentiated areas (2). Altered p53 protein expression, indicative of p53 mutation, was considered when there was either a strong accumulation within the nucleus (scored as focal or diffuse if there were <10% or >10% of positive cells, respectively) caused by prolonged half-life of p53 mutants (p53 mutated) or absence of p53 staining (3) within a context of WT staining (metaplasia or native epithelium [4]) caused by p53 mutations, leading to truncation or epigenetic silencing, in which case p53 was not detected by IHC (p53 negative [absent]). Bars: (main images) 100 µm; (insets) 20 µm. **(B)** Histogram showing the summary of p53 status in metaplasia and dysplasia areas of each case analyzed.

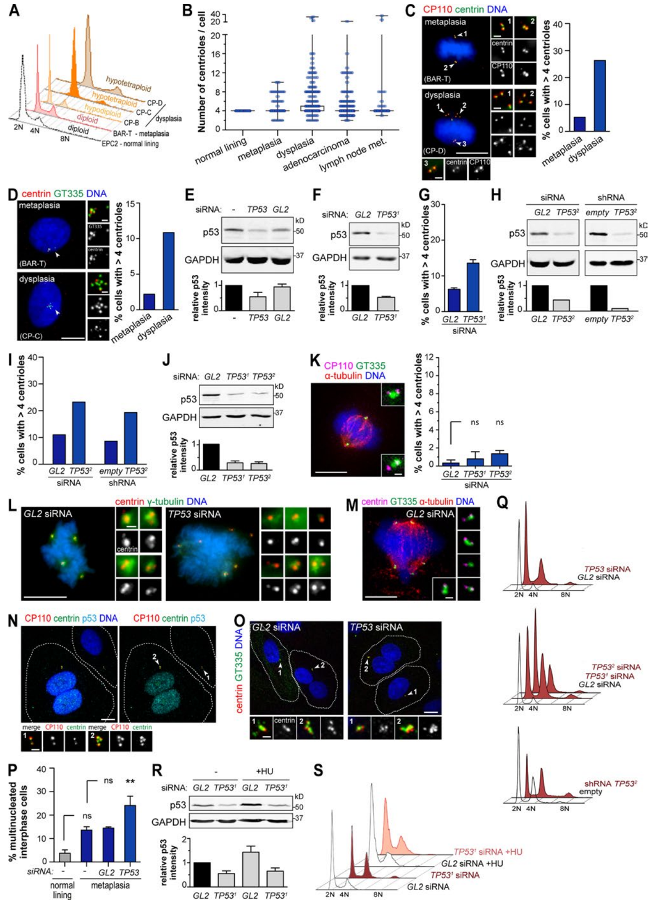

Figure S3. **Centriole number and ploidy in cell lines.** **(A)** Flow cytometry analysis of cells derived from the normal lining, metaplasia, and dysplasia. **(B)** Cells derived from the normal lining and from all stages of BE progression were stained for centrioles (centrin and GT335) and DNA. The number of centrioles was analyzed in each individual mitotic cell (individual circles, $n \geq 60$/cell line) of the indicated tissue of origin. Individual data points are plotted over the box plots. Met., metastasis. **(C and D)** Left: Metaplasia and dysplasia cells were stained for centrioles (centrin and CP110 in C, and centrin and GT335 in D) and DNA. Representative images with enlargements of centrioles (arrowheads) are shown. **(C)** Right: Quantification of mitotic cells with centriole amplification. $n \geq 50$/cell line. **(D)** Right: Quantification of mononucleated interphase cells with centriole amplification. $n \geq 90$/cell line. **(E)** Metaplasia cells were transfected with control (*GL2*) siRNA or siRNA against endogenous p53 (*TP53*). Top: Depletion of p53 was confirmed by WB. Bottom: Quantification of p53 relative to the loading control (GAPDH) is shown; ratios were normalized to the untreated cells. Three independent experiments. **(F and G)** Metaplasia cells were transfected with control (*GL2*) or p53 (*TP53*^{1}) siRNA. **(F)** Top: Depletion of p53 was confirmed by WB. Bottom: Quantification of p53 relative to the loading control (GAPDH) is shown; ratios were normalized to the control. Two independent experiments. **(G)** Cells were stained for centrioles (centrin and GT335) and DNA. Quantification of mitotic cells with centriole amplification. $n \geq 60$ cells/condition/experiment. Two independent experiments. **(H and I)** Centriole number was analyzed in metaplasia-derived cells containing pSUPER-p53RNAi (shRNA *TP53*^{2}) or the empty pSUPER vector (*empty*) as well as in metaplasia cells transfected with control (*GL2*) siRNA or siRNA against p53 with the same sequence against p53 (*TP53*^{2}) as the one used to generate the pSUPER-p53RNAi plasmid. **(H)** Top: Depletion of p53 was confirmed by WB. Data are from one experiment. Bottom: Quantification of p53 relative to the loading control (GAPDH) is shown; ratios were normalized to the controls. **(I)** Cells were stained for centrioles (centrin and GT335) and DNA. Quantification of mitotic cells with centriole amplification. $n \geq 100$ cells/condition. One experiment. **(J and K)** Normal lining cells were transfected with control (*GL2*) siRNA or two independent siRNAs against p53 (*TP53*^{1} and *TP53*^{2}). **(J)** Top: Depletion of p53 was confirmed by WB. Bottom: Quantification of p53 relative to the loading control (GAPDH) is shown; ratios were normalized to the control. Three independent experiments. **(K)** Left: Cells were stained for centrioles (CP110 and GT335), microtubules (α-tubulin), and DNA. Representative images with enlargements of centrioles are shown. Right: Quantification of mitotic cells with centriole amplification. $n \geq 80$/condition/experiment. Three independent experiments. **(L and M)** Metaplasia cells transfected with control (GL2) or p53 (*TP53*) siRNA were stained for centrioles (centrin), PCM (γ-tubulin), and DNA (L) or for centrioles (centrin and GT335), microtubules (α-tubulin), and DNA (M). Representative images with enlargements of centrioles are shown. **(N)** Metaplasia cells were stained for p53, centrioles (centrin and CP110), and DNA. Dashed lines denote individual cell outlines given by the CP110/centrin background signal. Insets show centrioles (arrowheads) in p53-negative (1) and p53-positive (2) cells. **(O and P)** Metaplasia cells transfected with control (*GL2*) or p53 (*TP53*) siRNA were stained for centrioles (centrin and GT335) and DNA. **(O)** Dashed lines denote individual cell outlines given by the centrin/GT335 background signal. Insets show centrioles (arrowheads) in mononucleated (1) and multinucleated (2) cells. Bars: (main images) 10 µm; (insets) 1 µm. **(P)** Quantification of multinucleated interphase cells. Untreated metaplasia and normal lining cells were also analyzed. $n \geq 700$ cells/condition/experiment. Three independent experiments. **(K and P)** **, $P < 0.01$ (ANOVA). **(Q)** Flow cytometry analysis of metaplasia cells transfected with control (GL2) siRNA or three independent siRNAs against p53 (endogenous p53, *TP53*; total p53, *TP53*^{1} or *TP53*^{2}) and of metaplasia-derived cells containing pSUPER-p53RNAi (shRNA *TP53*^{2}) or the empty pSUPER vector (*empty*). **(R and S)** Metaplasia cells transfected with control (*GL2*) or p53 (*TP53*) siRNA were either treated with (S phase blocked and hydroxyurea [+HU]) or without hydroxyurea (DMSO, asynchronous, and –). **(R)** Top: Depletion of p53 was confirmed by WB. Bottom: Quantification of p53 relative to the loading control (GAPDH) is shown; ratios were normalized to the control. Three independent experiments. Error bars show means ± SEM. **(S)** S phase blocking of cells upon hydroxyurea treatment was assessed by flow cytometry analysis.

Table S1. **Centriole number analysis in paraffin-embedded tissue**

**Tissue areas**	**Centriole number per cell (min–max)**				**Cells with centriole amplification**			
					%			
**Normal lining – esophagus**								
Cases 1–14	0–2				0.0			
**Normal lining – ileum**								
Case 1–14	0–2				0.0			
	**M**	**DYS**	**ADC**	**LNM**	**M**	**DYS**	**ADC**	**LNM**
**Cohort 1**								
Case 1	1–4	-	-	-	0.0	-	-	-
Case 2	1–3	-	-	-	0.0	-	-	-
Case 3	1–4	-	-	-	0.0	-	-	-
Case 4	1–4	-	-	-	0.0	-	-	-
Case 5	1–4	-	-	-	0.0	-	-	-
Case 6	1–4	-	-	-	0.0	-	-	-
**Cohort 2**								
Case 1	1–5	1–7	-	-	1.5	8.5	-	-
Case 2	1–5	1–5	-	-	1.5	4.0	-	-
Case 3	1–6	1–7	1–7	-	1.5	9.5	7.5	-
Case 4	1–72	1–76	1–6	-	3.5	9.0	5.0	-
Case 5	1–6	1–14	1–17	-	1.5	7.0	7.0	-
**Cohort 3**								
Case 1	1–5	-	1–8	-	0.5	-	3.0	-
Case 2	1–6	-	1–13	1–22	1.0	-	5.5	13.0
Case 3	1–4	-	1–7	1–7	0.0	-	0.5	2.5
Case 4	1–4	-	1–8	-	0.0	-	2.0	-
Case 5	1–4	-	1–7	1–8	0.0	-	1.0	6.5
Case 6	1–5	-	1–7	-	0.5	-	1.5	-
Case 7	1–4	-	1–12	-	0.0	-	2.0	-
Case 8[a]	1–5	-	1–5	1–15	0.5	-	2.5	9.5
Case 9	1–4	-	1–10	1–19	0.0	-	0.5	13.0
Case 10	1–7	-	1–5	-	2.5	-	6.5	-
Case 11	1–4	-	1–8	1–7	0.0	-	1.5	5.0
Case 12	1–5	-	1–8	-	0.5	-	3.0	-
Case 13	1–5	-	1–6	-	1.0	-	3.0	-
Case 14	1–4	-	1–5	-	0.0	-	2.0	-

ADC, adenocarcinoma; DYS, dysplasia; LNM, lymph node metastasis; M, metaplasia.
[a]The cell line ESO51 used in this study was derived from the primary tumor of this case.

Table S2. **Analysis of TP53 status**

**Case/cell line**	**Experiment**	**Metaplasia**			**Dysplasia**		
		**Coding**	**Protein**	**% Reads**	**Coding**	**Protein**	**% Reads**
**Patient tissue samples – cohort 2**							
Case 1	NGS	216delC	V73fs	22.5			
					586C>T	R196*	60.4
					569_570insC	P191fs	32.0
					86_87insA	N29fs	1.6
		751A>C	I251L	3.5	751A>C	I251L	4.4
		153_154insA	Q52fs	24.2	153_154insA	Q52fs	28.1
		215C>G	*P72R*	*60.9*	*215C>G*	*P72R*	*74.5*
	IHC	WT			Negative (absent)		
Case 2	NGS	n.d.			n.d.		
	IHC	WT			Intensity: moderate		
					>10% cells mutated		
Case 3	NGS				455delC	P152fs	96.6
					452C>A	P151H	3.4
		751A>C	I251L	3.0	751A>C	I251L	4.2
		404G>T	C135F	36.9	404G>T	C135F	49.9
		215C>G	*P72R*	*62.3*	*215C>G*	*P72R*	*76.5*
	IHC	Intensity: weak			Intensity: strong		
		<10% cells mutated			>10% cells mutated		
Case 4	NGS	733G>A	G245S	3.4			
					799C>T	R267W	17.6
					153_154insA	Q52fs	22.9
		751A>C	I251L	3.3	751A>C	I251L	6.0
		215C>G	*P72R*	*62.3*	*215C>G*	*P72R*	*76.5*
	IHC	WT			Intensity: strong		
					>10% cells mutated		
Case 5	NGS				638G>A	R213Q	3.3
					524G>A	R175H	6.6
					153_154insA	Q52fs	30.1
		215C>G	*P72R*	*51.5*	*215C>G*	*P72R*	*45.8*
	IHC	WT			Intensity: moderate		
					>10% cells mutated		
**Cell line**							
BAR-T	NGS	*215C>G*	*P72R*	*98.3*			

*, nonsense mutation; fs, frameshift mutation; n.d., not determined due to insufficient amount of quality DNA; text in italics indicate known polymorphism in p53.

Table S3 is an attached Excel file showing cell line information and centriole analysis.
